# When Small Words Foretell Academic Success: The Case of College Admissions Essays

**DOI:** 10.1371/journal.pone.0115844

**Published:** 2014-12-31

**Authors:** James W. Pennebaker, Cindy K. Chung, Joey Frazee, Gary M. Lavergne, David I. Beaver

**Affiliations:** The University of Texas at Austin, Austin, Texas, United States of America; West China Hospital of Sichuan University, China

## Abstract

The smallest and most commonly used words in English are pronouns, articles, and other function words. Almost invisible to the reader or writer, function words can reveal ways people think and approach topics. A computerized text analysis of over 50,000 college admissions essays from more than 25,000 entering students found a coherent dimension of language use based on eight standard function word categories. The dimension, which reflected the degree students used categorical versus dynamic language, was analyzed to track college grades over students' four years of college. Higher grades were associated with greater article and preposition use, indicating categorical language (i.e., references to complexly organized objects and concepts). Lower grades were associated with greater use of auxiliary verbs, pronouns, adverbs, conjunctions, and negations, indicating more dynamic language (i.e., personal narratives). The links between the categorical-dynamic index (CDI) and academic performance hint at the cognitive styles rewarded by higher education institutions.

## Introduction

The ways we use words reflect how we think. In trying to assess people's intellectual potential, common sense might dictate that we should pay attention to their use of long words or obscure references. The current study suggests that scholarly aptitude is better reflected in the ways people use short words. Following from previous literature showing how small word use reflects psychological states and cognitive processing, we applied computerized text analysis on a large corpus of college admissions essays with associated data on scholarship. The findings revealed how a single measure of word use correlated with future academic success. College admissions essays contain more clues to students' thinking styles than many scholars or administrators might guess.

Most universities require college admission essays in order to get a better sense of their applicants [1]–[2]. The underlying idea is that having prospective students write about their own experiences, interests, and goals can reveal something about the students themselves – the ways they think, their emotional states, and their general writing abilities. Ironically, there is little standardization in coding these dimensions. This is made even more difficult because applicants write on very different topics in different ways, making a standardized grading system challenging.

With the revolution of computerized text analysis, we can now start to determine which language dimensions in college admissions essays could be related to academic performance with an eye to understanding their underlying psychological or cognitive processes. There are several computerized essay-grading systems that assess content [3]–[4], and many more sophisticated natural language processing (NLP) tools and algorithms for classifying texts [5]–[6]. Virtually all of these tools focus on what people are writing rather than on the ways they write. An alternative way to explore people's writing styles is to focus on their use of function words using relatively simple word counting software programs such as Linguistic Inquiry and Word Count (LIWC [7]).

Programs such as LIWC calculate the percentages of words in any given text file belonging to previously categorized word categories. The word categories, or dictionaries, can be based on standard linguistic definitions, such as articles (a, an, the), or by agreement of independent judges [8]. Some of these categories include function or closed class words, which are the smallest yet most common words in the English language. Function words generally include pronouns, articles, prepositions, conjunctions, auxiliary verbs, negations, and many common adverbs.

It might be said that function, or closed class, words provide the bones for what we want to say, where content, or open class, words provide the meat. The closed class words connect, shape, and organize content, and have remained relatively fixed in the history of the English language; open class words express substantive properties of things and events in the world and so their relative appearance in daily language use changes with what is going on in an individual's world. There are further contrasts. While published dictionaries provide broadly agreed upon meanings for open class expressions, the exact meaning of even the most common function words (e.g. *the*, *a*, or *I*) remains controversial for scholars of linguistic semantics, pragmatics, and philosophers of language.

Across multiple studies using LIWC and other computerized text analysis methods, function words tend to be more reliable markers of psychological states than are content words such as nouns and regular verbs [9]. For example, high rates of pronoun use have been associated with greater focus on one's self or on one's social world [10], auxiliary verb use has been associated with a narrative language style [11], [15], article use has been associated with concrete and formal writing [12], and preposition and conjunction use has been associated with cognitive complexity [13]. Function words, then, can point to psychologically meaningful correlates of potential success in ways that are “invisible” to a human judge reading and coding admissions essays for higher-level constructs (such as achievement orientation, goal strivings, etc.). That is, function words allow us to assess how people are thinking more than what they are thinking about.

Against the background of this body of prior work demonstrating the efficacy of function words for establishing general traits of a speaker or writer, we now seek to establish more narrowly whether function word use can be predictive of scholarly aptitude, and potentially reveal general thinking styles reflective of academic success. To this end, we have linked function word use in a large corpus of college admissions essays with students' academic performance during their first four years of college. Three overlapping questions were addressed:

**Question 1.** Do function words and their presumed underlying cognitive styles predict later grade point average (GPA)?

**Question 2.** To what extent does function word use vary across writing samples in a coherent manner, with use of words in different categories jointly contributing information that may meaningfully be combined in a single, underlying dimension?

**Question 3.** Do function words improve the predictive accuracy of GPA models based on high school performance and college aptitude tests?

## Methods

### Measurement and psychometrics of function words

Although function words can be categorized in slightly different ways, the current project focused on eight broad dimensions as measured by the computerized text analysis program, LIWC: personal pronouns (e.g., *I, her, they*), impersonal pronouns (*it, thing*), auxiliary verbs (*is, have*), articles (*a, an, the*), prepositions (*to, above*), conjunctions (*and, but*), negations (*no, never*), and common adverbs (*so, really, very*). The LIWC word lists were compiled from multiple sources including grammar texts [8] and lists of commonly misspelled words (*hes* for *he's*) or writing shortcuts (*alot* for *a lot*). A complete list of the approximately 370 function words making up each LIWC category is available at https://utexas.box.com/s/9ncte8lmq5s1xemw3q1x. Generally, function words in LIWC are assigned to a single category. Exceptions include contractions (e.g., *I'm* is assigned to both personal pronoun and auxiliary verb categories).

LIWC analyzes each text separately and calculates the percentages of total words accounted for by each of the eight function word categories. As seen in Table 1, the mean percentage of articles in the admissions essays was 6.8% of the total words used. Note that the LIWC analyses resulted in one set of function word percentages per essay (recall that each student wrote two essays). Comparison data on function word frequency from a range of corpora is available at http://tinyurl.com/odr9tb9.

**Table 1:** Usage rates of LIWC's Function Word Categories in the Admissions Corpus.

Function Word Category	Examples	Rate of Use (%)	*SD*
Articles	*a, an, the*	6.83	1.30
Prepositions	*all, below, much*	14.71	1.41
Personal pronouns	*I, us, you, hers, they*	10.88	2.05
Impersonal pronouns	*it, this, anything*	5.03	1.38
Auxiliary verbs	*are, did, have*	8.25	1.72
Adverbs	*even, just, usually*	3.90	1.04
Conjunctions	*and, so, until*	6.41	1.02
Negations	*No, never, not*	1.04	.49

Note: Rate of Use refers to the percentage of total words that each function word category was used over the entire sample.

### The admissions essay corpus

The corpus of admissions essays was made up of more than 50,000 essays from 25,975 applicants who enrolled into a large state university as first year students from the years 2004 and 2007. A single text file was prepared for each of the over 50,000 essays.

In addition to the essays themselves, the university provided demographic data from the students' applications (e.g., sex, age, parental education, etc.). On average, applicants were 17.9 years old (*SD* = 0.42), 53.5% were female, and 92.1% were classified as in-state students. Although over 7,000 new undergraduate students enrolled each year, admissions were selective with the average student's high school GPA being in the top 9.5% of their graduating class (or the equivalent of being in the 90.5^th^ percentile). All college entrance exams were converted to their Scholastic Aptitude Test (SAT) equivalence, ranging from 400 to 1600, with a mean of 1245 (SD = 156). The concordance was based on a very large population at the state university [14], not a “national” concordance developed by ACT and College Entrance Examination Board most often used by smaller institutions. The ethnic breakdown of the students across the four years was 54.1% white of European descent, 19.2% Asian American, 18.6% Latino/a, 4.8% African American, 0.4% American Indian, and 2.9% international.

When applying to the university, applicants were required to complete two admissions essays on two separate topics from a list of 6–8 topics that varied slightly by year. All topics were quite general, asking students to describe people or events that shaped their development and influenced their goals for the future. The average length of each essay was 558 words (*SD* = 195).

The GPAs ranged from 0.00 to 4.00, and were cumulative (i.e. based on all courses completed by students in their college courses at each year), and were highly correlated across years. Note that that the sample sizes for available years of GPA vary for a number of reasons (i.e. not every college student completes four consecutive years of college from the time of their acceptance). Only the first three years of GPA data were available for the 2007 entering class.

### Ethics Statement

The project was approved by the University of Texas at Austin Institutional Review Board (reference number 2008-12-0080) on February 9, 2009, and judged to be exempt from the informed consent requirement. The exempt status was based on the project's being archival educational research and on the fact that the data, supplied by the Admissions Office, were analyzed with all identifying information removed.

## Results

Using LIWC, rates of the eight function word categories were computed separately for each of the two essays from each student. Consistent with previous research [13], the rates of use of each of the function word categories were positively correlated with each other across the two essays, ranging between.22 and.40, averaging.28 (equivalent to a Spearman Brown reliability coefficient of.76). As depicted in Table 1, the percentages of each of the function word categories were averaged across the two essays yielding eight mean percentages for each participant. These averaged values across the two essays per participant were used for further analyses.

### The relationships among function words: the CDI

The eight function word categories represented a total of approximately 370 words and accounted for 57.1% (*SD* = 3.58%) of all words used in the essays (see Table 2). A principal components analysis on the eight dimensions yielded a single factor that accounted for 35.1% of the variance. As described below, the single factor was referred to as a categorical – dynamic index, or CDI. Although all eight function word categories loaded on a single dimension, two had positive loadings (articles, prepositions) and the remainder had negative loadings (personal pronouns, impersonal pronouns, auxiliary verbs, conjunctions, adverbs, and negations). For each person, a single standardized factor score was computed using the factor loadings. In addition, a simpler unit-weighted CDI was created:

**Table 2:** Function Word Pearson Correlation Matrix.

	Articles	Preps.	P.Pron.	I.Pron.	Aux. verbs	Adverbs	Conjunc.	Negat.
Articles	1.00	.250	−.564	−.324	−.375	−.365	−.281	−.228
Preps.	.250	1.00	−.317	−.193	−.300	−.222	−.174	−.248
P.Pron.	−.564	−.317	1.00	.056	.221	.211	.109	.162
I.Pron.	−.324	−.193	.056	1.00	.521	.319	.054	.226
Aux. verbs	−.375	−.300	.221	.521	1.00	.309	.089	.294
Adverbs	−.365	−.222	.211	.319	.309	1.00	.277	.198
Conjunc.	−.281	−.174	.109	.054	.089	.277	1.00	.047
Negat.	−.228	−.248	.162	.226	.294	.198	.047	1.00

*Note*. Preps.  =  prepositions. P.Pron.  =  personal pronouns. I.Pron.  =  impersonal pronouns. Aux. verbs  =  auxiliary verbs. Conjunc.  =  conjunctions. Negat.  =  negations. All correlations are statistically significant, p<.01, 25, 973 *df*.

CDI = 30 + article + preposition - personal pronoun - impersonal pronoun – auxiliary verb – conjunction – adverb – negation.

The reason for the unit-weighted CDI score was to construct a simple, transparent algorithm that could be applied to other samples. Note that the value 30 was added to the word percentages so that the resultant score was typically positive. The factor analytically derived component score from the single factor was highly correlated with the simpler additive model, *r*(25,973) = .98, allowing us to simply add the percentage of articles and prepositions and subtract the remaining six function word categories. The unstandardized Cronbach's alpha of the 8-item index was.71.

The component loadings, the unit-weighted CDI score, and the simple correlations among the function words paint identical pictures: there is an internally-consistent, bipolar index that bears a striking resemblance to related language distinctions in previous research. Examples of previously examined indices include informational (nouns) vs. involved (verbs, auxiliary verbs, and pronouns) production [12], [15]; non-immediate (articles and big words) vs. immediate (auxiliary verbs, and pronouns) language [13]; formal (nouns, adjectives, articles, and prepositions) vs. contextual (verbs, pronouns, adverbs, interjections) style [16], and categorical (nouns, adjectives, prepositions, articles, and conjunctions) vs. narrative (verbs, adverbs, and pronouns) thinking [17]. We find similar patterns: At one end of the distribution are essays that use high rates of articles and prepositions and, at the other end, essays that tend to have high rates of pronouns, auxiliary verbs, conjunctions, adverbs, and negations.

Closer inspection of essays high in the use of articles and prepositions revealed relatively formal and precise descriptions of categories (e.g., objects, events, goals, and plans). Essays high in the use of pronouns, auxiliary verbs, and other function words were more likely to reveal changes over time, typically involving personal stories. By definition, the more that students used articles and prepositions, the less likely they were to use pronouns and other function words and vice versa.

This Categorical-Dynamic Index, or CDI, is a bipolar continuum that can be applied to any type of text. Categorical language is a style that combines heightened abstract thinking (associated with greater article use) and cognitive complexity (associated with greater use of prepositions). A lower CDI involves a greater use of auxiliary verbs, adverbs, conjunctions, impersonal pronouns, negations, and personal pronouns. These word categories, particularly pronouns and auxiliary verbs, have been associated with more time-based stories and reflect a dynamic or narrative language style [12].

### Predicting academic performance with the CDI

Simple correlations between the summed CDI index and GPA were modest but highly significant, such that higher categorical language was associated with better academic performance across all four years of college: *r _year 1_*(25,561) = .20, *r_year 2_*(25,905) = .19, *r_year 3_*(25,906) = .19, and *r_year 4_*(18,681) = .18. Although modest in magnitude, the correlations are noteworthy. Unlike college entrance exams, the essays were undoubtedly written in different settings from person to person, likely reviewed by friends, family and teachers, and with most students not having any explicit training in function word use.

Consistent with the directions of factor loadings in the CDI index, the individual function word categories correlated significantly with GPA in the predicted direction across the four years of college. Only articles (mean *r* = .12) and prepositions (.04) were positively correlated with GPA. The remaining function words were negatively correlated with GPA: auxiliary verbs (−.21), impersonal pronouns (−.15), personal pronouns (−.10), adverbs (−.09), conjunctions (−.06), and negations (−.02).

Students apply for admission and are eventually accepted into one of the eleven undergraduate colleges (Architecture; Business; Communications; Education; Engineering; Fine Arts; Geology; Liberal Arts; Natural Science; Nursing; Social Work). Within each college, simple correlations between CDI and GPA were computed. The CDI-GPA correlations were all positive (*r*'s range.09 to.30). The CDI-GPA correlations were highly significant (*p*'s<.001) for all schools except for those with fewer than 200 students (i.e. Architecture CDI-GPA *r*(169) = .16, *p* = .03; the college in Geology that opened midway into our study CDI-GPA *r*(116) = .10, *p* = .31).

Together, the results suggest that categorical language is consistently linked with better academic performance, whereas dynamic language is not (see also [15]). Interestingly, these effects held across all colleges (e.g., Engineering, Fine Arts, Liberal Arts, Nursing, etc.) at the university.

### Comparing the CDI with traditional predictors of academic performance

As seen in Table 3, higher CDI was correlated with having higher college board scores, coming from parents with more years of education, being male, and graduating somewhat lower in their high school class. Note that this pattern of findings is similar to earlier findings that a more formal style (marked by high use of nouns, adjectives, articles, and prepositions, and a low use of pronouns, verbs, adverbs, and interjections) was used more by males relative to females, and by more educated individuals [16]. It is ironic that although males generally use greater categorical language, their mean college GPA is somewhat lower than that of females in our sample.

**Table 3:** Intercorrelations among Predictors of Academic Performance.

	CDI	High school percentile	SAT equivalence	Sex	Parental education	Mean college GPA
CDI	1.00	−.047	.245	−.108	.220	.196
High school percentile	−.047	1.00	−.042	.115	−.145	.182
SAT equivalence	.245	−.042	1.00	−.118	.448	.411
Sex	−.108	.115	−.188	1.00	−.038	.100
Parent education	.220	−.145	.448	−.038	1.00	.277
Mean college GPA	.196	.182	.411	.100	.277	1.00

*Note*: All correlations are significant, p<.01. Mean college GPA is the mean grade point for students across all their years of college. When applying for admission, students must take either the SAT (originally called the Scholastic Aptitude Test) or the ACT (originally named the American College Test). Because the vast majority took the SAT, all ACT scores were converted to the SAT equivalence. Higher Categorical-Dynamic Index (CDI) scores indicate a more categorical thinking style. For sex, 1 =  male, 2 =  female. Parental education is based on the mean number of years of parents' education. High school percentage is scored such that 100% would be at the top of the class.

Table 3 also includes correlations between the traditional predictors of academic performance and GPA. Although universities rely on somewhat different statistical models in predicting college GPA, most include college boards such as the Scholastic Aptitude Test (SAT) and high school class rank. A simple forced-entry linear regression on yearly GPA found that SAT equivalence score and high school rank yielded an adjusted R^2^ of 219 for year 1, .206 for year 2, .193 for year 3, and .184 for year 4. (Note that the R^2^ statistic refers to the total variance accounted for, where .219 is equivalent to 21.9 percent of all the variance). Adding the single CDI index from function word analyses of the admissions essays increased the adjusted R^2^ to .230 for year 1, and to .216, .203, and .193 for the remaining years. The single CDI index added about 1% of the variance each year. If the eight individual function word categories were forced into the equation instead of the overall index, the predictive model increased by about 2% of the variance each year.

A model that included sex and parental education increased the overall adjusted R^2^ to .244 for the first year and down to .237 for year 4. In all cases, the percentage added by the CDI or individual function word categories was identical to the increase obtained when they were added to the more limited model that included only SAT equivalence score and high school rank only: in each case there was an increase of 1–2 percent in explained variance.

On the surface, a 1–2 percent increase in the variance accounted for in academic performance may sound relatively trivial. An alternative way of thinking is that the simple counting of function words increase the percentage of variance accounted for from approximately 20 percent to almost 22 percent, which is a 5–10 percent improvement in the predictive model. Such an increment with a large sample hints at the power of the word analyses.

## Discussion

Previous studies have found that function word use reflects personality and a variety of social and psychological processes. As noted earlier, function word use has also been associated with cognitive thinking styles and psychological states. The current project extends this work by demonstrating that the ways prospective college students use function words in their admissions essays can foretell their academic performance for up to four years.

The most striking aspect of this project is that the most common and forgettable words in English can reveal the ways people think. Language is associated with observable behaviors that have implications for students' success and for researchers' understanding of that relationship. In the growing age of big data, we can now begin to identify the potential thinking patterns of individuals, groups, and perhaps even cultures for whom there exist language records. Rather than adopt a machine learning approach or capitalize on new data mining methods to maximize predictive models, our goal has been to explore a single language dimension that reveals one way that people think. Indeed, the discovery of the CDI raises several questions.

Can categorical thinking be trained? Those who naturally write in more formal and structured ways apparently come from family backgrounds and high schools that instilled this form of writing and thinking. To the degree that it is trainable, one could easily build a feedback system in writing classes that provided CDI scores. At the very minimum, the information about CDI could help individuals to think in a more formal, logical, and hierarchical way.

Should future admissions offices rely on word counts to decide who should come to college? Probably not. As soon as word got out, enterprising students would soon be taking function word training courses to game the system. Rather, it is important to explore what categorical thinking says both about the applicant and the university.

The findings raise questions about the degree to which categorical language styles are valued in American education [17], [18], [19]. Most exams and papers in college courses require students to analyze and categorize concepts in a formal way. The writing of stories or other narratives is far less common. Are our secondary and higher educational systems discouraging students from writing in more dynamic or narrative ways? To the degree that dynamic language can enhance or balance performance - academic or otherwise, future research should consider how its value can be recognized in how we define success.

## References

[1] Atkinson R (2001) Standardized tests and access to American universities. Am Council on Educ. Washington, DC. Available: http://works.bepress.com/richard_atkinson/36. Accessed 15 June 2012.

[2] Walker B, Ashcroft J, Carver LD, Davis P, Rhoes L, et al. (2012) A review of the use of standardized test scores in the undergraduate admissions process at The University of Texas at Austin: A report to President Larry R. Faulkner by Task Force on Standardized College Admissions Testing. Univ Texas Austin. Available: http://www.utexas.edu/student/admissions/research/taskforce.html. Accessed 15 June 2012.

[3] Landauer TK, Laham D, Foltz P (2003) Automated scoring and annotation of essays with the Intelligent Essay Assessor. Assess Educ 10:295–308.

[4] Zenisky AL, Sireci SG (2002) Technological innovations in large-scale assessment. Appl Meas Educ 15:337–362.

[5] Joachims T (2002) Learning to classify text using support vector machines: Methods, theory, and algorithms. Dordrecht, The Netherlands: Kluwer Academic. 205 p.

[6] Larkey LS (1998) Automatic essay grading using text categorization techniques. In Proceedings of SIGIR-98, 21st ACM International Conference on Research and Development in Information Retrieval: 90–95. New York, NY, ACM.

[7] Pennebaker JW, Booth RJ, Francis ME (2007) Linguistic Inquiry and Word Count (LIWC2007): A text analysis program. Available: LIWC.net. Accessed 06 Dec 2014.

[8] Pennebaker JW, Chung CK, Ireland ME, Gonzales AL, Booth RJ (2007) The development and psychometric properties of LIWC. Available: http://homepage.psy.utexas.edu/homepage/faculty/Pennebaker/reprints/LIWC2007_LanguageManual.pdf. Accessed 06 Dec 2014.

[9] Pennebaker JW (2011) The secret life of pronouns: What our words say about us. New York, NY: Bloomsbury Press. 368 p.

[10] Tausczik YR, Pennebaker JW (2010) The psychological meaning of words: LIWC and computerized text analysis methods. J Lang Soc Psychol 29:24–54.

[11] Jurafsky D, Ranganath R, McFarland RD (2009) Extracting social meaning: identifying interactional style in spoken conversation. In Proceedings of NAACL, 2009 Annual Conference of the North American Chapter of the Association for Computational Linguistics, Human Language Technologies: 638–646.

[12] Biber D (1988) Variation across speech and writing. Cambridge, UK: Cambridge University Press. 316 p.

[13] Pennebaker JW, King LA (1999) Linguistic styles: Language use as an individual difference. J Pers Soc Psychol 77:1296–1312. 10626371 10.1037//0022-3514.77.6.1296

[14] Lavergne GM, Walker B (2001) Developing a Concordance Between the ACT Assessment and the SAT I: Reasoning Test for The University of Texas at Austin. Austin, TX: University of Texas.

[15] Robinson RL, Navea R, Ickes W (2013) Predicting final course performance from students' written self-introductions: A LIWC analysis. J Lang Soc Psychol 32:481–491.

[16] Heylighen F, Dewaele JM (2002) Variation in the contextuality of language: an empirical measure. Found Sci 6:293–340.

[17] Nisbett RE, Peng K, Choi I, Norenzayan A (2001) Culture and systems of thought: Holistic versus analytic cognition. Psychol Rev 108:91–310. 10.1037/0033-295x.108.2.291 11381831

[18] Graesser AC, Whitten SN (2001) Scripts of the mind and educational reform. PsycCRITIQUES 46:261–262.

[19] Schank RC (1999) Dynamic memory revisited. New York, NY: Cambridge University Press. 316 p.

